# Marine Polymer-Gels’ Relevance in the Atmosphere as Aerosols and CCN

**DOI:** 10.3390/gels7040185

**Published:** 2021-10-28

**Authors:** Mónica V. Orellana, Dennis A. Hansell, Patricia A. Matrai, Caroline Leck

**Affiliations:** 1 Polar Science Center, Applied Physics Laboratory, University of Washington, Seattle, WA 98195, USA; 2 Institute for Systems Biology, Seattle, WA 98109, USA; 3 Department of Ocean Sciences, RSMAS, University of Miami, Miami, FL 33149, USA; dhansell@miami.edu; 4 Bigelow Laboratory for Ocean Sciences, East Boothbay, ME 04544, USA; pmatrai@bigelow.org; 5 Department of Meteorology, Stockholm University, 11419 Stockholm, Sweden; lina@misu.su.se

**Keywords:** marine gels, DOC, aerosols, CCN, SML, central Arctic Ocean

## Abstract

Marine polymer gels play a critical role in regulating ocean basin scale biogeochemical dynamics. This brief review introduces the crucial role of marine gels as a source of aerosol particles and cloud condensation nuclei (CCN) in cloud formation processes, emphasizing Arctic marine microgels. We review the gel’s composition and relation to aerosols, their emergent properties, and physico-chemical processes that explain their change in size spectra, specifically in relation to aerosols and CCN. Understanding organic aerosols and CCN in this context provides clear benefits to quantifying the role of marine nanogel/microgel in microphysical processes leading to cloud formation. This review emphasizes the DOC-marine gel/aerosolized gel-cloud link, critical to developing accurate climate models.

## 1. Introduction

Dissolved organic carbon (DOC) is the largest reservoir of reduced organic carbon in the world’s oceans (~662 Pg C; [1]), similar in size to the atmospheric reservoir of CO_2_ (750 Pg C [2,3]). Of autotrophic origin mostly, this enormous biopolymeric pool is consumed by heterotrophic bacteria and protozoa [4]. Applying the principles of soft matter physics to understand marine biopolymer dynamics, Chin et al. [5] demonstrated that marine biopolymers assembled into a distinct supramolecular organization forming microscopic 3-dimensional polymer-gel networks embedded in a solvent (seawater) and ranging in size from nanometers to microns [5]. Spontaneous assembly of marine polymer gels occurs in the oceans when a chemically heterogeneous, polydispersed mixture of organic biopolymers (proteins, carbohydrates, lipids, and nucleic acids) interacts to form randomly tangled 3D, cross-linked hydrated networks. These are held together by ionic bonds (Ca^+2^), hydrophobic bonds, hydrogen bonds, and/or van der Waal forces, with a characteristic assembly/dispersion equilibrium depending on the nature of the polymers and the relation with the solvent [5,6,7,8,9]; for details please see Verdugo [10] in this volume.

Marine polymer assembly is reversible, follows second/first order kinetics, and exhibits an in vitro approximate thermodynamic yield at equilibrium of 10% (25–30% in the central Arctic Ocean) in surface waters and lower in deep waters (3–5%); thus most likely, ~70 Pg C of DOC [9] remains in dynamic and reversible assembly equilibrium, forming porous networks [5,7] in a colloidal size continuum [9]. As such, marine polymer gels have been proposed to play a pivotal role in regulating ocean basin-scale biogeochemical dynamics [11]. Marine gels concentrate and accumulate in the sea surface microlayer [7,12,13], and are then available for air–sea exchange as organic aerosols, as observed in the central Arctic Ocean [7,13,14,15] and, potentially, as a source for cloud condensation nuclei (CCN) formation [7,15]. Marine primary (aerosols emitted directly into the atmosphere) organic aerosols are airborne nanometer size to micrometer size particles (liquid, two phase liquid, solid) in the atmosphere that have been additionally quantified over the Atlantic and the Pacific Oceans and other oceans as well [16,17,18,19]. Thus, understanding marine biopolymer dynamics is critical to developing accurate models of the response of oceanic and atmospheric biogeochemical cycles to climate change. One crucial area is understanding the role of marine biopolymer nanometer size gels in cloud formation, and the link between the ocean’s surface biology to the atmosphere and climate [7,19,20,21,22]. Understanding the gel’s sources, their emergent properties (assembly, volume phase transitions), composition, fluxes, and size distributions (among other characteristics) is necessary to assess their susceptibility to influence cloud formation processes.

**Figure 1:**
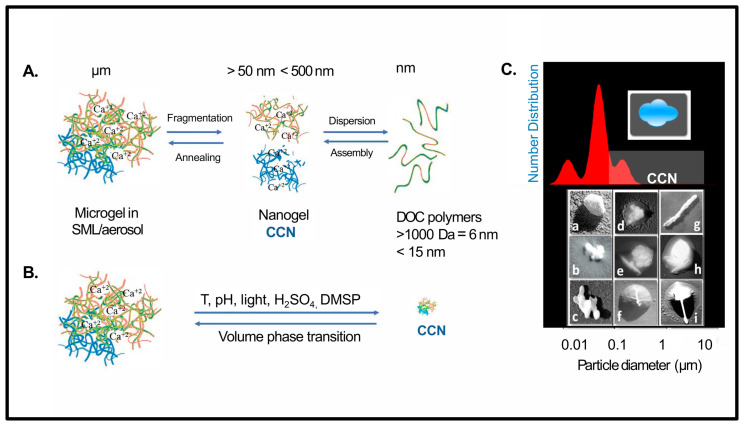
Conceptual figure indicating dynamic processes affecting gels as aerosols, CCN, and free polymers. (**A**) At the ocean–air interface in the surface microlayer, dissolved organic carbon (DOC) polymers assemble in a reversible process into microgels stabilized by entanglements and Ca^+2^ bonds and/or hydrophobic moieties. Microgels are then available for air–sea exchange as organic aerosols by diverse processes (bubble bursting/wind). Aerosolized microgels can fragment into smaller size nanometer-size gels by UV exposure, and/or dispersion, or other processes. If the nanometer size gels are activated, they can nucleate into forming CCN. Furthermore, nanogels can further disperse into DOC free polymers, (adapted from Verdugo, 2012). (**B**) Microgels can also attain nanometer sizes by undergoing volume phase transition induced by environmental conditions such as changes temperature (T), pH, light, H_2_SO_4_, DMSP, and DMS. These nanometer size gels may also become CCN, however, this route has not been proven yet. (**C**) TEM pictures of aerosol particles collected over the central Arctic Ocean north of 80°N. Examples of the changing nature of the high Arctic particles in different modal diameters: (**a**–**c**) sub-Aitken mode, (**a**) penta-hexagonal structure, crystalline and hydrophobic in nature assumed to be a colloidal building block of a polymer gel, (**b**) small polymer gel-aggregate forming a “pearl necklace” morphology possibly indicating hydrophobicity, according to Saiani et al. (2009), slightly covered with hydrophilic viscous but not gelling polymeric material “mucus,” (**c**) another particle example similar to b, (**d**–**f**) Aitken to small accumulation mode, (**d**) particle with a high sulfuric acid content and with a gel-aggregate inclusion embedded in a viscous non-gelling film of high organic content, (**e**) gel-aggregate, and a particle resembling a bacterium with a small aggregate attached to it, possibly detached from the larger one. The “bubble-like shaped particles” may indicate a possible recent injection to the atmosphere at the air–sea interface, (**f**) particle containing mainly ammonium sulfate and methane sulfonate, (**g**–**i**) large accumulation mode, (**g**) a bacterium, (**h**) sea-salt with an organic content only present at the rare occasion of high winds > 12 m s^−1^, (**h**) sea salt and a bacterium coated with an organic film and by the concentric rings typical of droplets of sulfuric acid.

This article introduces the critical role of marine gels as a source of aerosols and CCN in cloud formation processes, emphasizing Arctic marine microgels. While there are many studies and reviews about organic aerosols and cloud formation, understanding organic aerosols and CCN in the context of soft matter physics can provide clear benefits to quantifying their role in microphysical processes leading to cloud formation. Understanding the response of biogeochemical cycles to environmental forcing, and specifically the DOC –marine gel/aerosolized gel-cloud link, is critical to developing accurate climate models (Figure 1).

## 2. Background: Marine Gel Relevance as Aerosols

Our knowledge about clouds endures as a limiting factor in our understanding of the climate system and consequently in climate modeling [23,24]. Clouds only form when water vapor condenses. However, in the atmosphere water vapor needs a substrate onto which to condense on—tiny airborne aerosol particles known as CCN. Typically, CCN fall within the submicron size fraction, ~100 nanometers in spherical diameter. Depending on their properties and heights, clouds can either warm the underlying Earth surfaces by triggering a localized greenhouse effect or cool them by outwardly reflecting solar radiation. If CCN are limited and sparse, the resulting clouds will contain fewer and larger droplets [25]. Such clouds will reflect and scatter less sunlight radiation into space while blocking the escape of heat from Earth’s surface, causing it to warm [26].

When CCN are plentiful [27], countless fine droplets form; the resultant clouds will scatter additional light and become better reflectors, thus cooling the surface below. Anthropogenic particles are essentially absent in the central Arctic (>80° N), especially during summertime. Instead, biological sources of particles may dominate [7,15,21,28,29,30]. This “clean” air, with few CCN, makes the low-level stratocumulus clouds optically thin, with fewer but larger droplets. Because of the direct link between production of organic carbon—specifically marine gels by microorganisms—and CCN [7,15], climate change, ocean warming, and acidification may affect the microbiota’s diversity and activity, directly affecting the production of gels and, hence, aerosols and cloud formation [31]. Over the last decades or so, research extending the high Arctic findings to lower latitude oceans, has stressed the presence and enrichment of marine organic matter particles of submicron sizes in airborne aerosols and cloud water [13,15,18,20,22,28,32,33,34,35,36,37,38,39,40,41,42,43,44,45]. Although these organic aerosol particles are commonly described in terms of the chemical composition of the size-segregated organic components of the marine primary aerosols or the functional group composition (i.e., low molecular weight carboxylic acids, other humic-like substances) (e.g., [18,46]), they most likely are hydrated polymeric species and most likely form hydrated nanonetworks, or marine nanosized hydrogels, as they derive directly from marine dissolved organic matter.

Exopolymer-like particles in the atmosphere were first discovered by Bigg and Leck [15,28,45,47]. These authors recognized that these particles displayed the physicochemical characteristics of “marine gels”, polymer networks with emergent properties (please see below), which was confirmed by Orellana et al. [7]. This understanding followed from their studies of a possible link between cloud formation and biopolymer gels, then described as exopolymer substances (EPS) in the surface microlayer (SML) (<100 µm thick at the air–sea interface) in the high Arctic sea-ice leads [14,31,48].

Exopolymer-containing marine particles are also known as transparent exopolymer particles (TEP) when operationally defined as particles stained with Alcian blue, a cationic copper-phthalocyanine dye dissolved in acetic acid at pH 2.5 [49,50] that preferentially stains COO^−^ acidic and half-ester sulphate reactive groups of acidic polysaccharides [51] and uronic acids [52,53]; or stained with Coomassie blue (CB) for particles containing proteins. TEP and CB particles have also been measured in the SML [54] and atmosphere in the North Atlantic [55]. However, the fixation with acetic acid (TEP) or citric acid at low pH (CB) changes the macromolecular conformation and physical dynamics of the particles.

Charlson et al. (1987) evaluated the existing evidence at the time linking the gas dimethyl sulfide (DMS) (produced by microbial food web interactions from its precursor dimethylsulfoniopropionate (DMSP) to the production of CCN over remote marine areas. This challenging “CLAW hypothesis” [56] proposed that, in the surface ocean, DMS gas emissions by phytoplankton and their subsequent known oxidation products in the atmosphere—methane sulfonic acid, sulfur dioxide, and sulfuric acid—trigger cloud formation, in turn cooling the ocean surface. This cooling effect would, in turn, affect further emissions of DMS by changing the speciation and abundance, or both, of marine phytoplankton, thus establishing a negative or stabilizing feedback loop. High Arctic observations in the early 1990s confirmed that the intermediate oxidation products provided most of the mass for the CCN-sized particles observed over pack ice [57]. The source location of most of the DMS, though, was found at the fringe of the central Arctic Ocean, at the hospitable edges of the pack ice (or marginal ice zone), not in the central area of the Arctic Ocean [57]. The distribution suggested that winds carried DMS-rich air at the edges of the pack ice [29] towards the North Pole, and oxidation of the airborne DMS created extremely small sulfuric acid-containing particles. Theoretically, these particles would grow slowly by further condensation of the acids until they were large enough to serve as CCN. Surprisingly, sulfuric acid was not involved with the production of the small precursors of CCN [58], which was later also confirmed by Quinn and Bates [41]. Instead, the high Arctic observations in the mid-1990s showed that these small precursors were organic material and mostly particles resembling bacteria and nanometer- and micron- sized gels that were accompanied by other larger particles, such as fragments of diatoms [15,28,37,45,59]. Subsequently, Bigg et al. [48] detected large numbers (10^6^–10^14^ mL^−1^) of similar particles within the SML between ice floes.

During the summer in 2008, at 87° N [60], when the ice melt in the central Arctic Ocean was maximal, and the Arctic ice leads were most prevalent, marine polymer gel’s identification, characterization, and quantification were conducted in seawater, and, for the first time, also in the SML, fog, cloud, and aerosols [7]. This work confirmed that assembled nano-, micro-, and fewer macro-sized gels found in the SML were similar to earlier findings [15]. Orellana et al. [7] also found that the airborne microgels may have the chemical surfactant properties necessary to act as CCN. However, to behave as effective CCN, these particles must first reach a critical size (equal or larger than 50 nm [20]) and meet other physicochemical properties and energy constraints. Leck and Bigg [37,59] and Karl et al. [61] speculated that the primary marine gel would disintegrate under some circumstances, generating smaller sized particles, most likely due to ultraviolet (UV) radiation cleavage [62], or they might disperse under the different physicochemical conditions (temperature, pH, ionic strength) present in the atmosphere than in the seawater. However, polymer gels may also attain smaller sizes by undergoing volume phase transition [5], also quantified in the Arctic surface waters [7]. Furthermore, it was demonstrated that microgels could also carry DMSP similarly to phytoplankton secretory vesicles [63], or be the site for DMS condensation [36]. Orellana et al. [7] additionally demonstrated the presence of peptide amphiphiles as important characteristic components of such polymer nano- and micro-gels. However, Martin et al. [64] suggested that amphiphilic biopolymeric gels cannot uptake water vapor and form cloud droplets and activate as CCN, most likely due to the effects of surface partitioning on the lowering surface tension not being taken into account in their calculations [65]; instead, they concluded the sulfate fraction of the particles that dominated the CCNs, possibly trapped within the polyanionic matrix of the gel [63]. Similarly, Ovadnevaite et al. [16] suggested a dichotomous behavior for the primary mixed surface marine organic aerosols, most likely due to the lowering of the surface tension [66], and inhibition of water uptake by the hydrophobic surface of the organic-rich gel particles, as demonstrated experimentally [67]. Other measurements in the high Arctic have also suggested organic aerosols-like gels, as well as sea salt aerosols and older, long-range transported continental aerosols [30,68,69]. Recently, Baccarini et al. [70] demonstrated that frequent new particle formation, which could potentially lead to CCN over the high Arctic pack ice, is enhanced by iodine emissions, most likely produced by the microbiota [71]. Iodination of natural organic matter involves iodination of aromatic moieties of humic substances (HS) and proteins that could be aerosolized [72]. Organo iodine is very abundant in the ocean [73] and, in the Arctic Ocean, HS are more abundant in the winter than in the spring-summer months [74]. Perhaps, both iodine emissions and nanogels impact the microphysical properties of the clouds over the central Arctic Ocean [75]. In fact, while some fraction of the hypoiodous acid (HOI) production by marine diatoms can be volatile, another fraction may also react with seawater DOC polymers, thus constituting a critical mechanism to transfer this chemical to the atmosphere via aerosolized gels [71].

## 3. Composition and Controls on Microgel Formation and Bioreactivity

DOC is biopolymeric [4] and phytoplankton and bacteria are the main producers [76,77,78]. DOC is operationally categorized into three major fractions according to its apparent biological lability [79]. All ocean depths and geographical areas contain the very old, biologically refractory DOC (RDOC concentrations < 45 μM, with bulk radiocarbon ages of >6000 years, [80]); a semi-labile fraction that accumulates in the surface ocean and mixes towards the ocean interior (10–30 μM), and a labile fraction produced daily at the ocean surface by autotrophs and degraded swiftly by heterotrophs (hours, days, months). Gel forming EPS (colloidal and macromolecular size) in the Gulf of Mexico and the Atlantic Ocean have been shown to have modern radiocarbon ages [81].

Phytoplankton produces DOC [7,12,82] by diverse mechanisms [83], including direct release [84,85], mortality by viral lysis [86,87], apoptosis [88,89,90], degradation of particulate organic matter by microbes [91], and grazing [92,93]. Phytoplankton alone release ~10–30% of their primary production into the DOC pool by regulated exocytosis and/or cell death [90] in the form of microgels and free biopolymers [5,94], also known as secretions, organic surfactants, and exopolymers. These contain carbohydrates [95,96,97], peptides and proteins [98], lipids [99,100], and other metabolites [82,101,102]. The DOC pool, in turn, drives heterotrophic bacterial growth and marine ecosystem dynamics [103,104,105]. Furthermore, ~50% of bacterial production is also released into the DOC pool by viral burst and mortality [87], most likely releasing bacterial membrane porins [106,107], hydrolases [108], fatty acids and lipopolysaccharides [100]. Bacteria also release refractory short-chain compounds [109,110].

In the central Arctic Ocean, polymer gels are produced from the biological secretions of marine phytoplankton [111,112,113,114,115], bacteria and sea ice algae (reviewed by Deming and Young (2017) [116] and references there in), as well as from cell debris [117]. These polymers accumulate at the SML, the upper most layer of the ocean (10–1000 mm thick [118]), where they are available for aerosolization and, eventually, cloud formation [7,37]. These polymers are rich in polysaccharides [119] and macromolecules such as ice- binding proteins, nucleic acids, lipids, phenols, and flavones [120]. Bubble bursting in the SML transfers polymer-gels/aerosols into the atmosphere [18,118,121,122,123]. The SML thus has a crucial role in several biogeochemical cycles, such as the carbon cycle, the transfer of gases and aerosols to the atmosphere and climate-related processes [7,14,19,31,54,118].

Phytoplankton community composition exhibits seasonal changes influencing the DOC composition and abundance [119,124], and thus affecting the aerosol composition, reactivity, and particle growth [39]. In winter, the aerosols composition is dominated by sea salt (83%) and other inorganic compounds (non-sea salt SO4^2−^, metasulfonic acid (MSA), and low organic matter (5–15%) [39,125]. In contrast, organic matter dominates in aerosols during the spring bloom (40–65%) with a low percentage of sea salt and other inorganic compounds [39,125], as well as during the subsequent bloom collapse by viral infection [22,126,127]. Likewise, the chemical composition of simulated microgels strongly influences the activation capacity and growth of aerosolized microgels to act as CCN [20], linking the gel’s marine source to their atmospheric role.

The aerosol’s chemical composition influences the microscale physical processes in the atmosphere, and their capacity to produce CCN. Therefore, the aerosol’s composition has been characterized by different methods ranging from real-time aerosol mass spectrometers, aerosol time of flight mass spectrometer (ATOFMS), Fourier-transform infrared spectroscopy, single particle spectroscopy, as well as chemical fractionation and analysis among others. Frossard et al. [128,129] reviewed several studies and methods used to determine organic matter composition and particle size from natural and bubbler-generated, nascent sea-spray aerosols. However, it is not clear that the same population and type of organic particles were sampled by these different studies [130,131]. Furthermore, these studies took place at different places and different times of the year when the phytoplankton populations producing organic material were different.

Chemical composition strongly influences the hygroscopicity activation capacity and growth of primary organic aerosols to act as CCN, determined experimentally as hydrophobicity, volatility, changes in surface tension, surface charge, or solubility ([46] and references therein). This is critical, as the capacity of marine DOC polymers in the surface ocean to assemble into gel particles (see below) is also established by ionic bonds, polymer persistence length, and charge density, as mentioned earlier [5,9,11,62]. However, the chemical composition of dispersed aerosol components may be very different from that of assembled gel particles [7], which also exhibit emergent properties (i.e., reversible volume phase transition from a swollen, hydrated phase to a condensed and compact phase and vice-versa, see below) that arise from their interactions with seawater, or their control by environmental stimuli in seawater. These gel properties could also determine their characteristics as CCN.

Size-specific measurements show that aerosol composition as well as CCN activity vary with aerosol size [20,39,41] could dispersion of the gels during analysis produce this finding? However, over the Pacific and the Atlantic Oceans, marine organic aerosols dominate the nanometer sized aerosols composition [16,17,66,132]. Because mass spectrometers can only analyze <1 μm size particles, it is thought that nanometer sized dissolved carbon that is refractory and of old age dominates the composition of sea spray and primary aerosols [17,18,133]. Conversely, nanometer sized aerosols could be the result of cleaved, dispersed organic and labile micron sized DOC gels, and perhaps mixed with old refractory nanometer sized gels, and hence not completely old and refractory (19–40%), as shown by radiocarbon aging and by the thermal stability of the organic material [7,133,134]. Thus, this question remains to be revisited.

## 4. DOC and Gels: Assembly of Biopolymers

Chin et al. [5] applied the principles of soft matter physics to understand marine biopolymer dynamics, demonstrating that marine biopolymers assemble into 3D gel networks. As indicated earlier, spontaneous assembly of marine polymer gels occurs in the oceans when a poly-dispersed mixture of marine biopolymers interacts to randomly form tangled 3D cross-linked networks, held together by ionic bonds (Ca^+2^), and/or hydrophobic forces, hydrogen bonds, Van der Waals forces, depending on the nature of the polymers and the relation with the solvent (in this case seawater) [5,6,7,8,9]. Marine polymer assembly is reversible, follows second or first order kinetics depending on the composition of the polymers [5,6,7], and exhibits an approximate thermodynamic yield at equilibrium of 10% in open ocean waters. During this assembly process, biopolymers tangle and anneal to form nanometer to micrometer sized, porous networks in 48 h in laboratory conditions which remain in dynamic equilibrium [5,7,9], as part of a colloidal size continuum [9]. The yields of gel assembly depend on the polymer length (see below), composition (see above), charge density, and the presence of hydrophobic moieties [6,98]. In high Arctic surface ocean waters, DOC polymer assembly follows first order kinetics, with an approximate thermodynamics yield at equilibrium of 25–30%. The difference in the kinetics of polymer assembly between geographical regions arises from the composition of the DOC biopolymers. In the Arctic Ocean, the biopolymers are amphiphilic, with hydrophilic biopolymers containing hydrophobic moieties [6,98].

Assembled microgels accumulated in the SML are then available for air sea exchange as organic aerosols and potentially as a source of CCN [7] in the Arctic Ocean as well as in other oceans [18,22,42]. During cloud formation, aerosols containing polymers uptake large amounts of water, hydrating and swelling, most likely facilitating the production of microdroplets. However, the assembly of aerosolized and hydrated gels is yet to be quantified and it could likely explain not only the formation of microdroplets but also the production of secondary marine organic gel particles in aerosols [37].

## 5. Microgel Size and Stability: Dependency on Polymer Length

Polymer theory states that the probability of assembly of polymers into polymer gels, their equilibrium size and their stability as tangled networks once assembled, increases with the square of the polymer length [135,136]. DOC polymers of greater length are able to assemble into stable and larger size polymer gel networks because the interactions between the polymer chains tangles (i.e., ionic, van der Waals, hydrophobic forces, etc.) and interpenetrations become stronger. However, nanometer sized (<1000 DA) molecules are either unable to assemble or they assemble into unstable and colloidal size networks due to the low degree of interaction between the polymer chains; thus, polymer length constitutes a central control on the primary organic aerosol ultimate size spectrum and on the necessary characteristics for being CCN (≥50 nm). This theory has multiple implications for cloud dynamics, and offers insights on the sources and sinks of cloud particles [11,62,135,136,137].

As stated above, it has been demonstrated that the assembly of polymer gels in 0.2 µm-filtered seawater exposed to UV-B radiation levels found in polar regions [138], or to bacterial degradation, was slower than in unfiltered controls [62,139]. These results suggest that photochemical or bacterial enzymatic degradation of polymers can drastically limit the supply of microgels of bigger sizes (>500 nm); UV-B-induced cleavage (in vitro and in the field) and biodegradation yield short-chain polymers that do not assemble or assemble into unstable colloidal (nanometer size) gels. However, proteins exposed to photooxidation and added reactive oxygen species at levels measured at the ocean surface and room temperature (22 °C) do not cleave into short polymers chains but instead aggregate, preserving the original proteins [140]. However, in Arctic waters at 87° N, microgels did cleave when irradiated with environmental levels of UV radiation and cold temperature (−2 °C, −4 °C [60,141]). Indeed, exposure to environmental levels of UV radiation resulted in a factor of three reduction of the marine microgel yield, indicating cleavage and dispersion of the gels. While reactive oxygen species where not measured in the Arctic Ocean experiments, the difference in ambient temperature (22 °C and −4 °C) may explain the different processes taking place in both of these measurements [7,140]. In fact, polar phytoplankton and bacterial species develop antifreeze proteins with distinct structural and antioxidant properties, as well as amino-acid composition [142], than the proteins (i.e., fetuin, bovine serum albumen, cytochrome c) tested by Sun et al. [140].

Polymer gels range from nanosized networks to a few microns [7,15] in the central Arctic Ocean. Small nanometer sized aerosol gels, which are the most abundant, can result from degradation and dispersion during long travel times over open waters, or over the pack ice that, when combined with freshly-produced long aerosolized DOC polymers, facilitate the simultaneous production of multi-sized aerosol particles [37,59,61], rather than the traditionally-observed chemical and/or condensational progressive particle growth in the atmosphere. UV-B radiation can also disperse already assembled gels when crosslinked polymers are degraded and cleaved by UV-B [62].

## 6. Volume Phase Transition: Effects of pH, DMS, and DMSP on Gel Dynamics

The assembly and dispersion of assembled macromolecules are also affected by environmental forcing, such as temperature [9,143], pressure, pH [5,7], DMSP and DMS concentration [7], polycations such as polyamines [144], trace metals [145], pollutants [146], electric fields [147], and light [148]. Changes in the previous parameters stimulate polymer gel volume phase transitions [149] (swelling or dehydration and condensation of the polymer gel networks) and further the collapse of microgels into a dense polymeric nanometer network. In the ocean, volume phase transition of polymers gels may increase the sedimentation rate of the gels into deeper [5,7,12,150], thus removing them as a source of aerosols at the surface. During gel dehydration and collapse, small molecules can be entrapped in the gel network at high concentrations, such as DMSP [63] or even proteins (RubisCO) that can be found unaltered in the deep ocean [150]. Volume swelling/dehydration of polymer gels may be an important process in the central Arctic Ocean, where nanomolar concentrations of DMSP and DMS as well the products of DMS oxidation, such as sulfuric acid, induce phase transition of the polymer gels in vitro [7].

New data indicating the presence of HIO having a role in new particle, and possibly CCN, formation [70] should be tested for a predictable phase transition of micron sized gels. Arctic Ocean microgels could also entrap iodine and its products [71], as they do for DMS [63]. We also predict Arctic Ocean aerosolized polymer gels to be sensitive to changes in temperature [9], where supercool temperatures in the clouds [141] could induce volume phase transition of polymer gel networks. Changes in temperature can also change the hygroscopic properties of the polymer gel network due to the induced phase changes of the polymer network within the spray drop, and thus facilitate the formation of rain drops. However, this must be explored and demonstrated.

Furthermore, the presence of trace metals in the central Arctic Ocean [151,152,153] could also induce gel volume phase transition.

## 7. Summary of Present Knowledge: Marine Polymer Gels as CCN in the Central Arctic Ocean

During the Arctic Summer Cloud Ocean Study (ASCOS 2008) study at 87° N [60], when melt was maximal and leads were most prevalent, identification, characterization and quantification of marine polymer gels in seawater and also in the surface SML, fog, cloud, and aerosols were performed [7]. These polymer gel networks reached high concentrations in seawater (10^6^–10^9^ mL^−1^), with assembly yields averaging 25–30%, which were higher than published previously [5,62,154]. Their sizes ranged from a few microns to nanometer size, with nanometer size polymer networks being the most abundant [7]. A specific fluorescently labeled antibody probe developed against in situ seawater and SML biopolymers confirmed for the first time that the particles found in the atmosphere (aerosol/fog/cloud) originated in the surface sea water, including the subsurface and SML [7]. The polymeric particles were likely released by the abundant sea-ice diatoms (*Melosira arctica* and *Fragilariopsis cylindrus*), other phytoplankton and bacteria, and behaved as nano- and micron size gels, demonstrating a direct link between CCN and microorganisms and, more specifically, marine biogenic polymers or marine gels [7,15]. The gel networks were held together by random entanglements and Ca^+2^ ionic bonds as well as by hydrophobic moieties. The gels comprised as much as 50% of the total dissolved organic carbon in surface waters and the SLM, and they assembled at 4 °C faster than previously observed, following first order kinetics, most likely due to the presence of hydrophobic moieties enhancing polymer assembly [6,155]. The marine gels also underwent volume phase transitions induced by DMSP as well as DMS, another indication that those particles displayed the physicochemical characteristics of gels. Gel abundance in seawater also correlated with enrichment of proteins containing hydrophobic amino acids (leucine, isoleucine, phenylalanine, and cysteine) and of DMSP in the SML [7]. The aggregates found in the SML were confirmed to be similar to earlier observations [15,48].

The surface activity of aerosol particles, specifically the effect on surface tension reduction and its effect on the equilibrium spherical radius of an aqueous drop, can significantly influence the cloud droplet forming ability of these particles. In an attempt to reduce some of the uncertainties surrounding the observed CCN properties promoting/suppressing cloud droplet formation over the Arctic pack, Leck and Svensson [20] used Köhler theory and simulated the cloud nucleation process using an adiabatic air parcel model that solves the kinetic formulation of water condensation on aerosol. They took advantage of highly size resolved impactor samples of inorganic water-soluble aerosol-bulk chemistry together with size-resolved electron microscope aerosol particle data collected on previous expeditions [7,15,36,45,156,157]. This simulation made possible a highly size resolved best “guess” of the unexplained particle number fraction assumed to consist of organic water soluble, slightly water soluble and non-water-soluble proxy constituents. The general conclusion from the simulations was the increase of a hydrophobic character, with decreasing diameter, of the activated particles. This suggested a hydrophobic character for the central Arctic Aitken-mode (15–80 nm diameter) aerosols that would in turn impede water uptake and suppress cloud activation below 0.4% water supersaturation. As such, it seems that very high water supersaturations (>0.8%) would be required in order for the Aitken mode particles to be activated. It is possible that such high water supersaturations occur where small total droplet number concentrations are present such that excess water vapor is not depleted by larger particles and helps sustain the cloud even when the Aitken particles have low hygroscopicity [158].

The results from the above studies are consistent with the dichotomous behavior [16] of the 3D structure of the polymer gels during cloud droplet activation. Initially, only partial wetting and only weak hygroscopic growth would occur since only part of the CCN surface exhibits strong hydrophilicity. Given time, the strong surfactant property of the gel hydrophilic entities would decrease surface tension, which would lead to a decrease in water vapor supersaturation necessary to promote cloud droplet formation [137].

Not only could the polymer gel surface facilitate nucleate cloud droplets, but it is possible that the protein amino acid sequences [159] may play an active role, i.e., through their ice binding and ice nucleating properties. Amino acids, such as phenylalanine, leucine, isoleucine, and cysteine, were enriched in the SML [7,14]. Peptides containing some of these amino acids are known to assemble into hydrogels [159] that may make them good ice nuclei (IN) [160], and are thus important to be accounted for as controllers of high Arctic mixed phase clouds. In fact, many polar planktonic microbes, such as the diatom *Fragilariopsis cylindrus* [161] and the prokaryote *Colwellia*, are known to express antifreeze proteins [162]. Identifying the amino acid sequences that provide the hydrophobic and the hydrophilic surface-active properties of the proteins in the gel supramolecular assembly would thus elucidate a critical step that can alter cloud reflectivity. Furthermore, protein to carbohydrate ratio has been shown to be directly related to the relative hydrophobicity or “stickiness” of the EPS [163], thus protein enriched microgels at the air-water interface could provide a “glue” in the aerosols and thus, aid in the CCN formation.

Although airborne nanogels may have the chemical surfactant properties necessary to act as CCN, to behave as effective CCN they must first reach a critical size (≥50 nm) and meet physicochemical properties and energy constraints of the system [20,46]. Leck and Bigg [37,59] and Karl et al. [61] speculated that the primary marine gels would fragment [46] and disintegrate under some circumstances, generating progressively smaller particles, most likely due to UV radiation cleavage [62]. That this happened is suggested by the similarity in the shape of the size distributions of air and water [15] with microcolloidal size aggregates < 70 nm diameter. The measured modal diameters were 30 and 50 nm, respectively, shifting the airborne distribution to smaller sizes and being consistent with the hypothesis that fragmented gel aggregates may form almost all the aerosol particles between ca. 15 and 80 nm diameter [59]. Indeed, on average, during five weeks spent in the high Arctic pack ice region during 2001 [46,57,59], surface microlayer-derived particles represented more than half the collected airborne submicron particles and, on all days, dominated the aerosol population below 70 nm diameter. The fragmentation of marine gel particles is a process that may also be governed by repeated condensation and dissipation of fogs or clouds, following the strong indication of fog-related aerosol source mentioned in Heitzenberg et al. [164].

However, it is not yet clear whether the polymeric material reached smaller sizes due to cleavage caused by UV radiation, or due to reversible volume phase transition induced by poorly understood stimuli at this time, due to dispersion of the gels during their time travelling as aerosols, and/or perhaps by a combination of all three factors, as has been described for other marine gels [5,7,62]; thus, this remains an open research question. Furthermore, atmospheric gels could also provide sites for condensation of the biogenic gas DMS and/or its oxidation products, which can form an appreciable part of the total high Arctic aerosol [156]. In 2005, Leck and Bigg [15] detected marine organic—presumably microgel—material in half or more of their aerosol samples, which were also coated with sulfuric acid.

The co-occurrence of atmospheric organic material and biologically active marine waters has, since the mid-1990s, been confirmed for the high Arctic, but it has also now been documented for temperate waters [18,37,47,165], more recently reviewed by Quinn et al. [19]. In the emerging picture of the Arctic atmosphere, DMS concentrations will determine the mass of the particles by producing material for their growth. However, it is the number of airborne gels, or primary particles that will primarily influence the number of CCN and the resulting optical properties of the cloud droplets. Indeed, research during the past two decades—reviewed by Leck and Bigg [58] followed by Quinn and Bates [41]—does not corroborate the CLAW hypothesis for remote marine regions.

The transport of marine precursors to primary aerosols is usually a function of sea-spray, resulting from wind generated bubbles, or other bubble sources, bursting at the air–sea interface [35,42,130]. The presence of sea-ice in the central Arctic Ocean has long deterred and/or minimized the presence of sea spray. Indeed, Leck and Bigg [59] and Leck et al. [13,45] had hypothesized that the source of gels found in clouds was the open water between ice floes and that those particles would be transferred to the atmosphere by the bursting of air bubbles at the air–sea interface. However, it was only in 2011 that bubbles in the central Arctic Ocean were found at lower wind conditions (wind speeds < 5 m s^−1^) during clear sky days and ice melting to have similar size spectra to other oceans, although smaller sizes predominate [166], and provided a likely mechanism for getting SML-material airborne. The particles were mainly organic in nature, but their sea-salt component increased at high wind speeds > 12 m s^−1^ and dominated both mass and number. Interestingly, Nilsson et al. [167] showed that particle flux from the leads coincided with days of low winds, which also included many of the days with nucleation. It is now known that the diatom *M. arctica*’s “mucous matrix” traps oxygen bubbles produced during photosynthesis [168]. Thus, one can anticipate that these bubbles may burst, transferring biopolymeric material into the atmosphere. Since *M. arctica* is a ubiquitous diatom, forming 3–80% of the total phototrophic biomass in Arctic waters and being the most productive phytoplankton accounting for 90% of the primary production in the region [168], such oxygen bubble bursting from the mucous matrix of *M. arctica* may be an important, yet seldomly a quantified process [168]. However, the source(s) and abundance of these bubbles remain elusive to determine; during the most recent icebreaker expedition in summer of 2018 Arctic Ocean MOCCHA (Microbiology-Ocean-Cloud-Coupling in the High Arctic), the unexpected presence of small cap waves within the leads appear to dominate as a source of bubbles with respect to other possible sources (e.g., release from sea ice, photosynthesis, upward transport) [H. Czerki, pers. comm.].

## 8. Conclusions: Gels as a Source of CCN

The high Arctic studies of primary aerosol organic polymer gels derived from the SML between sea ice leads, performed over the last two decades, have been expanded to other marine areas [18,19,37,165,169]. These studies show strong similarities in the morphological and chemical characteristics of the particles in all regions [15,21,47]. Most of the studies have focused on size-segregated chemical characterization of marine aerosol particles [18,35,39,42,129,132,170,171] that, more recently, were reviewed by Quinn et al. [19,172]. They all have suggested marine DOC at the ocean surface to be the source for these aerosol particles. However, they have not examined nor quantified gel-specific, physical-chemical characteristics and their emergent properties (assembly, phase transition, and the effect of physico-chemical characteristics of the environment that determine the gel’s pathway during assembly) of this sort of dissolved-to-particulate organic matter. Because this material has been confirmed to be DOC-based, these hydrated polymeric particles must be gels [5], and their emergent properties can provide insights into the processes controlling cloud formation, linking biology at the ocean surface with cloud properties. However, climate change and ocean acidification will increase temperature and lower pH that may, in turn, synergistically reduce the assembly of DOC polymers, as shown experimentally at temperatures applicable to tropical and subtropical areas [173], with significant effects on carbon cycling in the oceans, as well as affecting the production of aerosols and resultant cloud formation. We expect changes in the yield of the assembly in the Arctic as well, but this needs to be determined.

Gel-specific characteristics provide physico-chemical processes by which such particles can change their size spectra in a way that, when linked to aerosolization functions (i.e., sea spray, wind speed, sea surface temperature) (e.g., Salter et al. [174]) and marine DOC concentrations, would allow their parameterization into climate models.
